# Human enhancement through the lens of experimental and speculative neurotechnologies

**DOI:** 10.1002/hbe2.179

**Published:** 2019-10-14

**Authors:** Wessel Teunisse, Sandra Youssef, Markus Schmidt

**Affiliations:** ^1^ Biofaction KG Vienna Austria

**Keywords:** art–science, DIY, EEG, human enhancement, motivation, neurohacking, neurotechnology, normalization, speculative design, tDCS

## Abstract

Human enhancement deals with improving on and overcoming limitations of the human body and mind. Pharmaceutical compounds that alter consciousness and cognitive performance have been used and discussed for a long time. The prospect of neurotechnological applications such as brain‐steered devices or using invasive and noninvasive electromagnetic stimulations of the human brain, however, has received less attention—especially outside of therapeutic practices—and remains relatively unexplored. Reflection and debates about neurotechnology for human enhancement are limited and remain predominantly with neurotech engineers, science‐fiction enthusiasts and a small circle of academics in the field of neuroethics. It is well known, and described as the Collingridge dilemma, that at an early stage of development, changes can easily be enacted, but the need for changes can hardly be foreseen. Once the technology is entrenched, opportunities and risks start to materialize, and the need to adapt and change is clearly visible. However, carrying out these changes at such a late stage, in turn, becomes very difficult, tremendously expensive, and sometimes practically impossible. In this manuscript, we compile and categorize an overview of existing experimental and speculative applications of neurotechnologies, with the aim to find out, if these real or diegetic prototypes could be used to better understand the paths these applications are forging. In particular, we will investigate what kind of tools, motivations, and normative goals underpin experimental implementations by neurohackers, speculative designers and artists.

## 1 INTRODUCTION

Neurotechnology becomes ever more accessible (Dubljević, Saigle, & Racine, 2014; Wexler, 2016), not only for medical applications such as prostheses (Dubljević et al., 2014; Wexler, 2016) and psychiatric treatment (Deuschl et al., 2006; Loo et al., 2012), but also for nonmedical, even do‐it‐yourself (DIY) use (Wexler, 2016, 2017) and human enhancement (Dockery, Hueckel‐Weng, Birbaumer, & Plewnia, 2009; Egner & Gruzelier, 2003; Fregni et al., 2005; Meinzer et al., 2014). In 2019 the cheapest electroencephalograph (EEG) based brain‐computer interface (BCI) was available for just under USD 100 (NeuroSky, 2015a), and more advanced—as well as slightly more expensive—models can be downloaded and 3D printed (OpenBCI, 2019). This makes neurotechnology very accessible for the do‐it‐yourself community or neurohackers. While EEG is just one example within a wide range of tools that can be used for neurotechnology, the decreasing costs and increasing availability of devices—including g.tec since 1999 (UnicornBI, 2019a), NeuroSky since 2004 (NeuroSky, 2019), Emotiv since 2011 (Emotiv, 2019), OpenBCI since 2014 (Crunchbase, 2019), and Muse since 2014 (O'Rourke, 2015)—as well as the increasing number of available applications (as shown in this paper), lead to questions about the motivations behind DIY use and projects. An overview is relevant for both policymakers who want to regulate the DIY use in order to control the risks at an early stage (see Collingridge dilemma [Worthington, 1982]), and for companies that sell neurotechnology and therefore need to understand their customers' needs. To get a better understanding of the directions that neurohackers' and designers' neurotechnology projects are taking, this paper examines current applications of neurotechnology, categorizes them and focuses specifically on applications and concepts that surpass or move beyond pure medical or therapeutical purposes.

While neuroscience generally attracts considerable public interest, this is particularly true because of the rapidly expanding discourse on the *merging of human corporeality with technology*. In this discourse, *neurotechnology* often features as the harbinger of a future in which the body is transformed in a process of ever‐increasing “technologization.” It is expected that such *technologization* will happen in two ways:

1. Invasive: technological modifications of the body in which surgical interventions allow technology to replace or augment bodily functions (*implants*, *prostheses*).
2. Noninvasive: the use of technologies that modify the body without such interventions (*noninvasive neurostimulation technologies*), and *brain‐machine interface technology* capable of coupling humans and artifacts.

## 2 TOOLS

With the wide availability of neurotechnology tools for DIY use such as electroencephalography (EEG) based brain‐computer interfaces (BCI) and transcranial direct current stimulation (tDCS), the possible applications are more and more only dependent on the available or programmable software and, of course, imagination. Sites such as http://github.com provide a wide range of free downloadable software codes to use and many websites provide tutorials on how to use neurotechnology tools (NeuroTechX, 2019). Therefore, even people without any sophisticated background in neurology or computer science can start to use these applications at home.

An EEG headset consists of one or multiple electrodes placed on the scalp, a ground electrode and a reference electrode. The electrodes measure the changes in voltage potential created by the ion current within the nerves of the brain. More electrodes allow more detailed measurements. Simple headsets can measure the level of general focus or relaxation of an individual and show this as biofeedback. If the focus surpasses a defined threshold, one could couple an automated action to it such as extinguishing a candle (Chierico, 2014). Other biofeedback systems are based on the visual cortex: if the wearer of the EEG looks at a screen with blinking lights, the frequency of the blinking can be traced in the brainwaves in the visual cortex, thereby determining what the wearer is focusing on. This can be used to select something on a screen with multiple different options (displayed with different frequencies) to make decisions (Emmerson, 2018).

Where EEG headsets measure brain activity, transcranial direct current stimulation (tDCS) is an example of a technique that allows one to influence/stimulate brain activity. tDCS uses at least two electrodes placed on the scalp that apply a small current (0.5–2 mA) through the brain, which can be used for a wide range of effects, some of which are still poorly understood (Dubljević et al., 2014). This technique is closely related to transcranial alternating current stimulation (tACS).

There are other neurotechnological tools including transcranial magnetic stimulation (TMS), functional magnetic resonance imaging (fMRI), magnetoencephalography (MEG) and spinal cord stimulation. However, to our knowledge, they are, mostly due to the costs and sometimes regulatory hurdles, currently not available to neurohackers.

If the thresholds of access and accessibility are lowered, tools and applications can be appropriated by individuals from a variety of fields. This can be observed, for example, in the use of neurotechnology in art and art–science projects at the annual Ars Electronica Festival in Linz, Austria, an event well known for gathering artists, designers, and scientists to experiment, explore and innovate (ARSElectronica, 2019).

## 3 METHODS

To ensure broad coverage of scientific research, artists' work and DIY use of neurotechnology, a large number of different sources and databases were used for this work. First, all the exhibitions from the Ars Electronica festival (2013–2018) were examined in search of neurotechnology related projects, as Ars Electronica is the largest and most prestigious media arts festival, frequently linking art and science in unusual ways. Second, http://vimeo.com, http://newness.com and http://youtube.com were used to find art and design projects around neurotechnology. http://vimeo.com showed the best and most results, which were narrowed down by selecting videos that were posted no longer than 4 years ago, and which seemed to be an art–science project or neurotechnology product. Furthermore, the first two pages of the category tabs “Art & Design” and “Animation” were checked. Some of the videos were the outcome of actual scientific research, which often led to peer‐reviewed papers. DIY use was found by snowball sampling through different forums such as http://www.reddit.com and http://learn.neurotechedu.com. The latter houses a long list of examples of neurotechnology. These examples led to other projects that were related or functioned as inspiration. Often, DIY projects referred to scientific work to back‐up or substantiate their claims.

In addition, Google Scholar was used to research neurotechnology techniques and related technosocial models (such as Collingridge's dilemma and Technology Adaption Lifecycle) covered in this article. Research into neurotechnology techniques also opened up the existence of additional applications. The majority of the peer‐reviewed articles we found was in the medical field; however, as they were often purely medical we decided to discard them based on a lack of enhancement opportunities. The reference sections of the peer‐reviewed articles were searched for additional articles. Finally, the websites of the applications and tools showed us the scientific research they based their claims upon.

Because of the wide range of different platforms, the sources include peer‐reviewed paper, forum posts, online videos, books, blog posts, portfolios and news articles. The search terms were: future body, futurebody, neurotechnology, brain machine, brainmachine, human enhancement, mind uploading, mind machine, brain machine interface, brain computer, brain technology, brain implant, brain technology fiction, neuron, neurology, neurology fiction, neuron fiction, nootropics, technology biology, braintechnology, braincomputer interface, enhanced senses, transhuman, neuroenhancement, neuroprosthetics, posthuman, identity, life extension, eugenics, human hybrid, gene therapy, Neuroethics, brain prosthetics, BCI, Deep TMS, Transcranial magnetic stimulation, TMS, Deep brain stimulation, Ear‐EEG, Whole brain emulation, WBE, Neuromodulation, pulsed electromagnetic field therapy, PEMFT, neurostimulation, sacral nerve stimulation, spinal cord stimulator, SyNAPSE, Artificial brain, Transcranial direct current stimulation, tDCS, cortial modem, and Hippocampus prosthesis. This resulted in our finding and cataloging 56 neurotechnology applications further discussed below.

### 3.1 Applications

To get a comprehensive overview of neurotechnology in society we examined the identified applications considered in projects that use neurotech. Based on the search results we have grouped the applications into five categories of which we examined three in greater detail. The five categories are:

1. Medical
2. Med+
3. Enhancement
4. Speculative, and
5. Science Fiction.

The category “medical” encompasses neurotech applications and methods that have clear cut diagnostic and therapeutic purposes. On the other end of the spectrum, “science fiction” refers to extremely speculative or even unrealistic applications, barely grounded in technological possibilities or even surpassing the laws of physics.

The remaining three categories are of most interest for the scope of this manuscript. First, a quasi‐medical category is defined, which is called **Med+**, because applications in this group have originally had a medical approach or purpose, but also have potential to go beyond healing or restoring functions; they can allow enhancement of specific functions (e.g., hearing ultrasound). The second category, called **Enhancement**, considers applications that are nonmedical, but specifically serve other purposes, such as enhancement of senses or interactions with the environment. The third category is based on research and art projects that are not yet realizable and is therefore called **Speculative**. It points to applications that might occur in the future, but includes work based on current research or prototypes; this category encompasses scientific research (SR) as well as speculative design (SD).

The three categories can further be subdivided into invasive and noninvasive technologies. See Table 1 for an overview of the number of cases identified. In general the number of cases found for noninvasive applications clearly surpass the invasive types.

**Table 1:** Overview of neurotechnology applications beyond pure medical applications, and not in the realm of science fiction (*n* = 56)

	Med+	Enhancement	Speculative
Invasive	5	4	4
Noninvasive	9	19	15

## 4 RESULTS

Based on our online and literature search we identified 56 applications in the three categories Med + (13), Enhancement (22) and Speculative (21) (see Table 1). The following section will give an overview of the identified applications and highlight a few representatives and/or outstanding cases in all of the three categories.

### 4.1 Med+

Where there are many medical applications of neurotechnology available, the Med + category only include medical applications that have a prospect to provide enhancement over normal human capabilities (see Table 2). Often this is found in the restoration and improvement of senses such as hearing and sight. Since the first invention of cochlear implants in the 1960s, and seminal developments in the 1970s, cochlear implants have now become increasingly used (Mudry & Mills, 2013), restoring hearing in deaf patients. In the early 2010s, this is followed by a retinal prosthesis called Argus II (SecondSight, 2013), which (partly) restores vision for retinitis pigmentosa patients (Weiland & Humayun, 2014). These are both examples of technically advanced medical implants that restore lost functions. However, such implants might in the future also be used for enhancement, allowing the individual future patients of a visual prosthesis, for instance, to see in more detail or in frequencies outside the spectrum normally available to humans (e.g., infrared or ultraviolet).

**Table 2:** Identified neurotechnology applications in the category Med+

Invasive	Noninvasive
– Cochlear implant; device that translates sounds to electric pulses to directly stimulate the cochlear nerve (House, 1976)	– Unicorn Horn: Device that measure concentration can record moments of loss of focus (UnicornBI, 2019b)
– Retinal Prosthesis (Luo & da Cruz, 2016)	– Controlling a virtual avatar by BCI for disabled (UnicornBI, 2019a)
– Hear colors; The color blind Neil Harbisson has a device that allows him to hear different tones for different colors (Jeffries, 2014)	– Increase learning for learning disabled children (Fernández et al., 2003)
– Controlling a prosthetic arm by thought (Levy & Beaty, 2011)	– Rehabilitation for stroke patients (Kober et al., 2015)
– Controlling a robotic arm by thought for paralysis (Chadwick et al., 2011)	– Determine consciousness of people in coma/locked‐in syndrome (Guger et al., 2017)
	– Decrease stress and increase sleep (Tyler et al., 2015)
	– Paralyzed influence music by brain waves (2018)
	– Spell by thinking/watching the Unicorn Speller (Ortner, Aloise et al., 2011; Ortner, Prueckl et al., 2011)
	– Controlling an orthosis (BR41N.IO, 2019)
	– Biofeedback for meditation (Bhayee et al., 2016) (SR)

The cochlear implant, for example, does partially restore hearing loss, but can under specific circumstances, such as in a very noisy environment, endow the patient with better than human hearing capabilities, for example, when the microphone and software of the cochlear implant can filter out human voices from loud background noise.^1^

One prominent example of neurohacking is cyborg artist Neil Harbisson. Born completely color blind, Harbisson implanted an antenna into his head in 2004 that translates colors to sounds that he hears through bone conduction. This device partly restores his color vision, but also allows him to “see” (or rather: hear) color such as infrared and ultraviolet light (Jeffries, 2014) that are not part of the spectrum visible to humans. Implanting an experimental device such as the cyborg antenna is not something that is easily done: Harbisson was rejected by bioethics committees before he found, “*a doctor willing to do the surgery anonymously, and [he] found one in Barcelona*.” (Gartry, 2015). Technically speaking, Harbisson's Cyborg Antenna is not a brain implant, since the antenna is integrated in the skull. But we felt it would be a good addition, as the device allows Harbisson to hear colors in his head, opens up a sense he had been missing, and allows him to transcend what that sense could usually perceive (CyborgArts, 2016; CyborgArts, 2019).

Invasive applications such as the Cyborg Antenna or the cochlear implant are, however, minority options, and for the most part, we found noninvasive examples in the Med + category. The Unicorn Speller software, for instance, to be used together with an EEG headset is one of those applications available for consumers and often used at the BR41N.IO hackathon. Originally designed for use in the medical field, for example, for locked‐in patients, it can be used to spell words by watching a screen and counting the moments a specific letter or symbol blinks. This counting can be registered via the p300 paradigm by the EEG, and the correlation with the blinking of the icon on the screen allows the software to determine which symbol the users want to choose (Ortner, Aloise et al., 2011; Ortner, Prueckl et al., 2011). This is a rather slow approach to communication, but useful for people unable to speak or move. Another example is Alessio Chierico's interactive installation “Trataka,” wherein the user wears a BCI device and is asked to focus on a flame. An airflow is directed to the flame, and once the user puts full attention on it, it is extinguished. This principle could be used as feedback for meditation. Many EEG products supporting meditation practices are commercially available (Muse, 2018; NeuroSky, 2015a), which is backed up by scientific findings (Bhayee et al., 2016; Davidson et al., 2003; Lomas, Ivtzan, & Fu, 2015).

A technique using blinking frequency and synchronization in the visual cortex captured by EEG (as explained in the Tools – EEG section) allowed motor‐impaired patients to participate in making music with a string quartet (Emmerson, 2018).

The Cybathlon organized by the ETH Zürich has several disciplines that connect gaming and medicine. An example is a race where paralyzed persons have to control the artificial electrical stimulation of their legs with their thoughts (Azevedo Coste et al., 2017; ETH‐Zürich, 2019b; Laubacher, Aksoz, Bersch, & Hunt, 2017). This is done in order to literally cycle a trail. Other disciplines involve the control of prostheses, exoskeletons, wheelchairs or virtual avatars.

The use of EEG is also found in Anouk Wipprecht's Unicorn Horn. This is a device that is made for children that easily lose their attention in class. A light in the horn tells the teacher and the student the level of focus, which allows the teacher to adapt the lesson to the capabilities of the student. Also, the horn can record what the wearer focuses on when attention is lost. This could provide suggestions on how to maintain focus for a longer time (UnicornBI, 2019b).

tDCS is often used by DIY users for a broad range of medical/enhancement therapy. This includes treating mania, obsessive–compulsive disorder (OCD), depression and anxiety, improved erotic experiences etc. (Reddit, 2019). Most of the applications have never been scientifically investigated.

### 4.2 Enhancement

Neurotechnology enhancement implants took off with Kevin Warwick's chip implant in 1998 (Warwick, 2019a). This chip, which was implanted in his arm, allowed him to open doors, turn on lights, and control other devices without touching or moving. Whereas the first chip was not yet coupled to his nervous system, a second chip array, implanted in 2002, was (Warwick, 2019b). This chip allowed him to, closely related to medical applications, control an electric wheelchair and a robotic arm. The bidirectional function of the chip allowed Warwick to feel stimulations from the chip. This was demonstrated by the fact that he and his wife, who also had a chip integrated into her nervous system, were connected through their respective chips. In the following years, implants became wider available and cyborg communities, such as the Cyborg Foundation founded by Neil Harbisson (discussed above in the section dealing with Med + applications) and Moon Ribas, started promoting neurotechnology implants (CyborgFoundation, 2019). Cyborg artist Moon Ribas is known for her implants, which are connected to online seismographs and which resulted in giving her additional senses that allowed her to “see” movement behind her back and to feel earthquakes all over the world (CNN, 2018; Garcia, 2015; Quito, 2016).

Among the most accessible implants we find the insertion of magnets, for example in the tip of a finger (Robertson, 2017). This allows the individual to feel magnetic forces and also control magnets or magnetizable objects. DIY YouTube videos are available to teach interested parties how to implant a magnet by themselves (TheThoughtEmporium, 2017).

Closely related to implanting magnets is Cyborg Nest's “North Sense,” a device pierced into the chest that vibrates when the wearer is facing north. The mission statement of this self‐titled “Mindware Company” is to, “contribute to human evolution. By experiencing the hidden parts of nature through new senses, we will evolve towards a richer life experience.” (CyborgNest, 2018).

Apart from implants, be they integrated into the nervous system or not, there are many noninvasive examples to enhance human capabilities. One of the main interests seems to be gaming. Many innovations focus on the control of avatars in video games: for instance, in the virtual race called “Cybathlon” (ETH‐Zürich, 2019a; Riener, 2016), or in the commercially available “Adventures of Neuroboy” by Neurosky (NeuroSky, 2015b), and by many games produced during the BR41N.IO hackathon, a 24 hour long BCI challenge event staged in various places across the world multiple times per year since 2017, in which teams come together to develop BCI applications (BR41N.IO, 2019).

One practical possible future application arising from the hackathon is the control of home products also referenced under the phrase “Smart Home.” This application allows users to measure brain waves with an EEG headset and to put on any device including lights, a heater or an AI helper like Apple's Siri. The applications of the hackathon make use of the “Unicorn Speller,” which, as described previously in the section “Med+,” requires users to focus on specific icons flashing on a screen.

While companies such as the streaming service Netflix (2019) and some music video clips (Coldplay, 2014) are experimenting with user‐defined narratives—meaning that the user influences the way the story develops—, neurotechnology is doing the same, however, working with the unconscious. Rachel and Richard Ramchurn's “The MOMENT” project is a brain‐controlled film: Based on brain activity and blinking measured by EEG headsets, the film can be unconsciously influenced by some audience members (Pike, Ramchurn, Benford, & Wilson, 2016).

Much as Warwick and his wife Irena experienced a connection through their chip implants neurotechnology allows noninvasive ways to have such a connection, such as the control over somebody else's limbs. Greg Gage, who co‐founded the neuroscience company Backyard Brains (BackyardBrain, 2019) which sells educational and DIY neuroscience kits for schools and neurohackers, demonstrated on of his kits on stage: At a TED talk, he asked two participants on stage and applied electrodes to their arms. One of them could move the other's arm. This is done by measuring the electrical current in the first person's arm, while they are moving it, and applying a correlated electrical current to the second person's arm (Gage, 2015). While this connection uses electrodes on muscles, as early as 2013, researchers at the University of Washington achieved a human‐to‐human brain interface using EEG on the sender's end and transcranial magnetic stimulation on the receiver's end (Armstrong & Ma, 2013).^2^

Finally, we find another method in enhancements: transcranial direct current stimulation (tDCS). Closely related to the tDCS applications for medical purposes, tDCS is used in a similar way for enhancement. When used in the right way (and what exactly the right way is, is still up for debate), tDCS can lead to enhanced planning ability (Dockery et al., 2009), enhanced working memory (Fregni et al., 2005) and enhanced learning capacity (Meinzer et al., 2014). Users report they experience increased ease, fewer distractions and a decreased number of “background” thoughts (Adee, 2012). Most importantly for the purposes of this paper, tDCS devices are readily available and, ranging between USD 150 and USD 300—or building one yourself for just the material costs^3^—, and thus fairly accessible in terms of pricing, as well as reviewed in recommendation lists online (neurogalMD, 2018; TotaltDCS, 2019).

A list of examples of Enhancement applications can be found in Table 3.

**Table 3:** Identified neurotechnology applications in the category Enhancement

Invasive	Noninvasive
– Seismic Sense; allowing users to feel earth (CNN, 2018; Garcia, 2015; Quito, 2016)	– Improved vocabulary learning and maintenance by tDCS (Meinzer et al., 2014)
– Magnet in finger for magnetic feeling and magnetic capabilities (Robertson, 2017)	– Film narrative control based on brain waves (Pike et al., 2016)
– Control doors, lights, and other household devices (Warwick, 2019a)	– Increase attention and working memory by neurofeedback (Jiang, Abiri, & Zhao, 2017) or by tDCS (Fregni et al., 2005)
– Feeling magnetic north (pierced) (Thaddeus‐Johns, 2017)	– Increase creativity by neurofeedback (Gruzelier, 2014)
	– Increased learning of music by neurofeedback (Egner & Gruzelier, 2003; Waters‐Metenier, Husain, Wiestler, & Diedrichsen, 2014)
	– Stimulated percussion; stimulated muscle movement for rhythm (Ebisu et al., 2017)
	– Echolocating headphones (Chacin, 2012)
	– Show augmented information when focusing on an object on a heads‐up display (EEG) (Puzzlebox, 2012)
	– Active control of software/avatars and devices for video gaming EEG (Metz, 2017)
	– Passive control for games EEG (StarWarsScience, 2015)
	– Increase physical training (tDCS) (Huang, Deng, Zheng, & Liu, 2019; Park, Sung, Kim, Kim, & Han, 2019; Waters‐Metenier et al., 2014)
	– Control somebody else's arm/body by electrical pulses (BackyardBrain, 2019; Gage, 2015)
	– Sense atmospheric pressure (Muñoz, 2017)
	– Feeling speed of objects (Ribas, 2015)
	– Feeling movement behind you (Ribas, 2015)
	– Influence liquid (Smigielska & Cutellic, 2018) or flame by brain activity (EEG) (Chierico, 2014)
	– Tracking and influencing of dreams (DreamLab, 2018)
	– Increase sleep for athletes (Abeln, Kleinert, Strüder, & Schneider, 2014)
	– Self‐tracking (Swan, 2013)

### 4.3 Speculative

This section collects potential *future* applications of neurotechnology, that are perhaps conceptual, but nevertheless not located in the realm of science fiction. Many possible future applications are in fact based on preliminary research carried out by scientists (Angrick et al., 2018; Bhayee et al., 2016; Hampson et al., 2012; Kapur et al., 2018; Novich & Eagleman, 2015; Pais‐Vieira et al., 2013; Ramakrishnan et al., 2015), while others emerge from other fields, including art and design. Correspondingly, we include examples marked by “SR” for scientific research and “SD” for speculative design in Table 4.

**Table 4:** Identified neurotechnology applications in the category Speculative

	Invasive	Noninvasive
Animals	– Coupling rat to rat and ape to ape brains with brain implants. (Pais‐Vieira, Lebedev, Kunicki, Wang, & Nicolelis, 2013; Ramakrishnan et al., 2015) (SR)	
	– Neuroprosthesis to restore or repair cognitive function, for example under influence of drugs (Hampson et al., 2012) (SR)	
Humans	– Speech synthesis from ECoG data (Angrick et al., 2018) (SR)	– Additional senses to the body (Hertrich, 2019) (SD)
	– Neuralink: implantable brain computer or brain interface for humans. (Urban, 2017; Musk, 2019) (SR)	– Connect people with brainwave synchronization (Bevilacqua et al., 2019; Dikker & Oostrik, 2019) (SD/SR)
		– Kissing data from EEG; what does it mean for the kissers and observers (Lancel, 2019) (SD)
		– Authentication based on EEG data (Swaine‐Simon, 2017) (SD/SR)
		– AlterEgo silent speech (telepathy) (Kapur, Kapur, & Maes, 2018) (SR)
		Computational augmentation (Kapur et al., 2018) (SR)
		Internet access in your head, allowing for a large amount of information directly augmented into your thoughts (Kapur et al., 2018) (SR)
		Cheating in games (Kapur et al., 2018) (SR)
		Control devices (Kapur et al., 2018) (SR)
		Feel changes in stock market (Eagleman, 2015) (SR)
		Feel sound/speech/language (Eagleman & Novich, 2019) (SR)
		Feel ISS quality (Eagleman, 2015) (SR)
		Feel twitter response (Eagleman, 2015) (SR)
		Feel airplane measurements (Eagleman, 2015) (SR)

Even though the possibilities of implants for DIY users at large are currently limited, the possibilities for self‐tracking or noninvasive applications seem endless. Another possible future application might be authentification: NeuroTechX examined the possibilities to use EEG data as a biometric for security purposes (Swaine‐Simon, 2017).^4^

In the realm of speculative art, “Kissing Data”/“E.E.G. Kiss” by Lancel and Maat (Lancel, 2019) examines the effects of kissing and watching people kiss in EEG data. To further explore the question of what it means to feel a connection with someone, cognitive neuroscientist and artist Suzanne Dikker explores the idea of synchronization of brainwaves in a series of science‐art projects. The methodology at the core of most of these projects encompasses measuring the brain waves of two persons through EEG data and asking them to actively try to synchronize their brain states. In one project this is done by dancing the tango, in two other projects people are allowed more liberty to find their own way of connecting (Dikker & Oostrik, 2019). This is also related to research done on the effect of brainwave synchronization between teacher and students on learning outcomes. It is shown that social closeness between teacher and student are of much higher influence on the learning outcomes than brain wave synchronization (Bevilacqua et al., 2019). Even more speculative is the concept of an installation designed by Susanna Hertrich over the span of several years titled “Prostheses for Instincts” that explores a device attached to the human body, which creates a new sense of awareness and emotional extension: The inputs vary from stock market data, currency exchange rates, natural disasters or crime rates, and a change of the data input (like a spike or drop) incites the device to react. The user then experiences sensations that are similar to physical reactions to immediate danger: goosebumps, shivers etc. (Hertrich, 2019). Hertrich's work is closely related to research done by David Eagleman that uses a haptic vest to input information into the back of the wearer (Novich & Eagleman, 2015). The research explores the possible information streams that our brains can learn to understand and feel as an additional sense. In a TED talk, Eagleman hypothesizes about how this could be used to get a feeling for multi measurement data, like all the airplane measurements for pilots, all quality measurements of the International Space Station (ISS) for an astronaut, or a common feel for changes in the stock markets. Due to the many variables influencing these measures it is difficult for humans to consciously analyze them, but the vest could give us a feeling for it that we might understand (Eagleman, 2015).

## 5 MOTIVATIONS

For each of the described categories (Med+, Enhancement, Speculative) different motivations, goals and normalization levels can be distinguished. The Med + category for both invasive and noninvasive technologies is marked by motivations to overcome mental or physical disabilities (Emmerson, 2018), for pain relief (Kapural et al., 2010), or, in fact, unraveling the mystery of consciousness (or unconsciousness) (Guger et al., 2017). But Med + devices can also work beyond the conventional medical uses—a cochlear implant can function as an aid to hear sounds, not within the usual human accesible frequency range, and the research put into a retinal prosthesis may well serve as a base for bionic eyes. The noninvasive devices in this category have also been first conceptualized from a medical or therapeutical standpoint, but hold the potential or actually can be used for other purposes.

On the border between Med + and Enhancement, we find health tracking, self‐optimization or self‐monitoring as the driving forces. Similarly to how users around the world are currently using apps and smart watches to track and self‐optimize their selves, these activities could (in the near future) also be done via invasive or noninvasive neurotechnology. These activities are mainly motivated by self‐control and self‐improvement (Wexler, 2017) or self‐actualization, and are mostly embedded in a competitive market economy environment.

The Enhancement category is defined by practical reasons, identity or self‐esteem factors, but also the motivation to change how life is experienced. Practical reasons are defined by goals that range from the general enhancement of human abilities and intelligence (Meinzer et al., 2014) to, alternatively, having to put in less effort (e.g., controlling the light or heater in your house) (Ammon, 2010). Another reason for Enhancement neurotechnology is for the sake of a gimmick or to perform a party trick (Robertson, 2017). This can be motivated by the desires to have fun, gain respect and attention or build self‐esteem, for instance. Here, neurotechnology gains attraction from people because of its futuristic elements. People might feel the desire to have the latest gadgets before anyone else (Thaddeus‐Johns, 2017). Others may wonder how the addition of extra senses will affect their perception and enjoyment of reality. They may want to discover the limits of human perception, and whether implants or additional senses can lead to new forms of expression, creativity or understanding in general. In the case of invasive technologies and implants, another possible consideration for individuals may be the desire to attain the next level of body modification—beyond tattoos and piercings (Thaddeus‐Johns, 2017).

Another group of distinguishable motivations is strictly found with DIY or home users of neurotechnologies. Similarly to what is seen in other fields of DIY science, these groups explore neurotechnology to democratize the tools of science, increase learning outcomes (Wexler, 2017), adapt the tools to their specific needs, and create applications without a commercial interest. While for the most part the intentions of the hackers are benign and constructive, they often lack in professional knowledge and skills, which can lead to some unintended self‐harm, for example when attempting to remove an implanted magnetic bead (aixre, 2017a, 2017b).

Finally, the category Speculative firmly points to the future, with speculative design concepts that explore possibilities. Some of the motivations overlap with motivations within the Enhanced group, such as the exploration of expression and potentials: additional senses (Hertrich, 2019), how it might “feel” to deal with multiple data inputs from unusual sources (Eagleman, 2015; Eagleman & Novich, 2019), what brainwave synchronization or interaction between two people may feel or look like (Dikker & Oostrik, 2019; Lancel, 2019). But the Speculative category also involves existing scientific research that serves as a basis for *potential* future applications. In the case of devices like AlterEgo, a wearable headset that can translate subvocalizations, or “silent speech,” and can also reply “silently” via bone conduction, the practical motivations are found in enhanced privacy and the fact that devices are strictly personal and not influenced by distance or background noises (Kapur et al., 2018). This consideration also applies to all implanted devices. And yet, the research, conducted at the Massachusetts Institute of Technology (MIT), also speaks to future applications that could come close to how we imagine “telepathy” (Kapur et al., 2018).

This is also the only category, which includes selected examples of animal research, because they contain a speculative dimension toward future potential human applications: specifically, animal experiments that couple rats or apes brain‐to‐brain (Pais‐Vieira et al., 2013); (Ramakrishnan et al., 2015), or that implant neuroprostheses in primates that can counteract drug influence (Hampson et al., 2012). These experiments may or may not see applications in humans, though given the fact that China is conducting a first clinical trial using experimental deep brain stimulation (DBS) on drug addicts with mixed reports, the topic seems of some relevance (Navarro, 2019). While DBS has been used successfully for several conditions, including Parkinson's disease, it is not fully understood. Regardless, in early 2019 the U.S. Food and Drug Administration has green‐lit a round of clinical trials to test the use of DBS for Opioid addiction (http://Clinicaltrails.gov, 2019). Whether DBS becomes a successful and legally sanctioned practice depends on the research results, bioethics committees and regulatory bodies. As a medical measure, once it has passed clinical trials and gained approvals, it could become a regular practice, and perhaps normalized.

### 5.1 Normalization levels

Yet, in this text, the neurotechnological applications discussed move beyond the purely medical, and are explored outside that field. The cases collected and categorized here are diverse and encompass both invasive and noninvasive technologies, goal‐driven as well as speculative applications. Whether or not these become normalized, is not easy to answer, as multiple disciplines field the issue of what a “social norm” is, or more specifically means, differently: in law social norms can be seen as something that is necessary to modify, in order to keep up with socio‐economic, environmental or technological changes (Spector, 2018). In psychology “normality” is frequently defined by juxtaposing it with “abnormality,” and diagnosis of the severity of that abnormality based on several criteria including statistical infrequency or violation of social norms (McLeod, 2018). Within Sociology what is “normal” is defined as collective or individual perceptions of acceptable conduct, largely dependent on social norms, which in turn, can often be endorsed separately or additionally in smaller groups, and are often situational dependent (Hechter & Opp, 2005; Schultz, Nolan, Cialdini, Goldstein, & Griskevicius, 2007). Instead of asking whether a certain technology is “normal” or not, or whether a neurohacker and cyborg is perceived as “normal” or not, it serves the scope of this paper better to reiterate what it takes for a technology to be “normalized.” Normalization process theory (or: NPT) is a sociological theory developed over the span of several years that originated in the healthcare system and is now used for science and technology studies. Its focus lies on how a new practice or technology becomes embedded in everyday life. It uses four constructs that deal with this: (a) Coherence—or the sense‐making people have to individually or collectively engage in; (b) Cognitive participation—or the work done to build and sustain a community of practice; (c) Collective action—or operational work, that is, how functional, integrated or workable a technology might be; and (d) Reflexive monitoring—or the reflection work that is needed to asses and understand how a new set of practices or technology affect them (May et al., 2015).

Neurotechnology captures a wide array of different technologies, goals, motivations and technology readiness levels (TRLs). While some of the cases explored here, specifically the scientific research with medical applications, may already be resident in coherence and participation, other cases illustrate forays into sense‐making.

Some of the examples covered here, such as the implants of sensors and chips, might perhaps be described by the word “gimmick.” This, by no means, suggests that these cases have no use. Apart from the crucial work of sense‐making and building a community of practice through such experiments, the first fun applications of basic research are in fact essential to overcome the trough of disillusionment within the Gartner hype cycle (Fenn, 2007)—which follows the peak of inflated expectations—by gathering enough early adopters as indicated by the technology adoption lifecycle (Rogers, 2003) for the further development of the field. These gimmicks are perfect examples of what kind of everyday applications can be expected in the near future. “Near future” is indeed appropriate for these applications, as they are already available and most often easy to use or program. The claim of “easy to use” is emphasized by the text inviting people to join one of the BR41N.IO hackathon events: “Anyone can participate who has interests in BMI (brain machine interface) […] Participants do not have to be a BMI expert to participate on a team!” (BR41N.IO, 2019).

### 5.2 Final thoughts

A study in the area of 3D‐printed weapons has shown how difficult it is to regulate DIY activities once published online (Bryans, 2015). To a large extent the same applies to the area of DIY neuroscience (and other DIY fields), as both practices are done at home and the materials are of everyday use.

In the near future, the influence of neurotechnology might increase due to the development and accessibility of the technology. Where some argue that by increased performance of wearables, invasive technology will become less popular (Robertson, 2017), other research shows that 8 out of 10 smartphone users envision a near future with implanted augmentation or body monitoring devices (internables) (Ericsson, 2015). Either way this would increase the use of neurotechnology and the need of more focus and care from users, developers, companies, and policy makers. Even though neurotechnology might have already been used for centuries (Sarmiento, San‐Juan, & Prasath, 2016), in most aspects it is still in its infancy. As described by Collingridge's dilemma of control, this is the time to shape the future of the technology (Worthington, 1982). It requires responsibility of its users and for developers to focus on their customers, for example, as outlined in the Responsible Research and Innovation (RRI) frameworks (Wickson & Carew, 2014).

## ENDNOTES

1 A patient with a cochlear implant told us that he considers himself a cyborg because of this enhanced hearing capabilities.

2 This work is continued in animal experiments listed in Table 4, coupling animal brain via brain implants.

3 However, we certainly do not encourage readers to try this at home.

4 Experience from the past, however, shows that it does not take very long for someone to hack a new biometric system, for example, fingerprints, retina scan, vene scans, and so forth. See also: https://media.ccc.de/v/35c3-9545-venenerkennung_hacken or https://media.ccc.de/v/biometrie-s8-iris-en

## References

[hbe2179-bib-0001] Abeln, V. , Kleinert, J. , Strüder, H. K. , & Schneider, S. (2014). Brainwave entrainment for better sleep and post‐sleep state of young elite soccer players—A pilot study. European Journal of Sport Science, 14(5), 393–402. 10.1080/17461391.2013.819384 23862643

[hbe2179-bib-0002] Adee, S . (2012, February 1). Zap your brain into the zone: Fast track to pure focus. *NewScientist*.

[hbe2179-bib-0003] aixre . (2017a). *Brutal magnet removal* Retrieved from https://forum.biohack.me/discussion/2038/brutal-magnet-removal‐

[hbe2179-bib-0004] aixre . (2017b). *Magnet removal pictures* Retrieved from https://docs.google.com/document/d/1cWFGHK5AVK-LNTZcGyBxvllfxLfDFPfCUhuckY3Aij4/edit

[hbe2179-bib-0005] Ammon, R. (2010). From event‐driven business process management to ubiquitous complex event processing. Retrieved from http://citeseerx.ist.psu.edu/viewdoc/download?doi=10.1.1.649.7584&rep=rep1&type=pdf

[hbe2179-bib-0006] Angrick, M. , Herff, C. , Mugler, E. , Tate, M. C. , Slutzky, M. W. , Krusienski, D. J. , & Schultz, T. (2018). Speech synthesis from ECoG using densely connected 3D convolutional neural networks. bioRxiv, 478644 10.1088/1741-2552/ab0c59 PMC6822609 30831567

[hbe2179-bib-0007] Armstrong, D. , & Ma, M. (2013). Researcher controls colleague's motions in 1st human brain‐to‐brain. Interface Retrieved from http://www.washington.edu/news/2013/08/27/researcher-controls-colleagues-motions-in-1st-human-brain-to-brain-interface/

[hbe2179-bib-0008] ARSElectronica . (2019). *ARS Electronica* Retrieved from https://ars.electronica.art/news/

[hbe2179-bib-0009] Azevedo Coste, C. , Bergeron, V. , Berkelmans, R. , Martins, E. F. , Fornusek, C. , Jetsada, A. , … Wolf, P. (2017). Comparison of strategies and performance of functional electrical stimulation cycling in spinal cord injury pilots for competition in the first ever CYBATHLON. European Journal of Translational Myology, 27(4), 251–254. 10.4081/ejtm.2017.7219 PMC5745381 29299228

[hbe2179-bib-0010] BackyardBrain . (2019). *Neuroscience for everyone* Retrieved from https://backyardbrains.com/

[hbe2179-bib-0011] Bevilacqua, D. , Davidesco, I. , Wan, L. , Chaloner, K. , Rowland, J. , Ding, M. , … Dikker, S. (2019). Brain‐to‐brain synchrony and learning outcomes vary by student–teacher dynamics: Evidence from a real‐world classroom electroencephalography study. Journal of Cognitive Neuroscience, 31(3), 401–411. 10.1162/jocn_a_01274 29708820

[hbe2179-bib-0012] Bhayee, S. , Tomaszewski, P. , Lee, D. H. , Moffat, G. , Pino, L. , Moreno, S. , & Farb, N. A. S. (2016). Attentional and affective consequences of technology supported mindfulness training: A randomised, active control, efficacy trial. BMC Psychology, 4(1), 60 10.1186/s40359-016-0168-6 27894358 PMC5127005

[hbe2179-bib-0013] BR41N.IO . (2019). *BR41N.IO at brainstorms festival 2019* Retrieved from https://www.br41n.io/Vienna-2019

[hbe2179-bib-0014] Bryans, D. L. (2015). Unlocked and loaded: Government censorship of 3D‐printed firearms and a proposal for more reasonable regulation of 3D‐printed goods. Indiana Law Journal, 90(2), 901–934.

[hbe2179-bib-0015] Chacin, A. C . (2012). *Echolocation headphones* Retrieved from http://www.aisencaro.com/echo.html

[hbe2179-bib-0016] Chadwick, E. K. , Blana, D. , Simeral, J. D. , Lambrecht, J. , Kim, S. P. , Cornwell, A. S. , … Kirsch, R. F. (2011). Continuous neuronal ensemble control of simulated arm reaching by a human with tetraplegia. Journal of Neural Engineering, 8(3), 034003 10.1088/1741-2560/8/3/034003 21543840 PMC3608269

[hbe2179-bib-0017] Chierico, A . (2014). *Trāṭaka* Retrieved from http://www.chierico.net/trataka/

[hbe2179-bib-0018] http://Clinicaltrails.gov. (2019). *Feasibility of deep brain stimulation as a novel treatment for refractory opioid use disorder (DBS OUD)* Retrieved from https://clinicaltrials.gov/ct2/show/NCT03950492

[hbe2179-bib-0019] CNN . (2018). *Moon Ribas: The cyborg dancer who can detect earthquakes* Retrieved from https://edition.cnn.com/style/article/moon-ribas-cyborg-smart-creativity/index.html

[hbe2179-bib-0020] Coldplay . (2014). *Coldplay—Ink* Retrieved from https://www.coldplay.com/ink/

[hbe2179-bib-0021] Crunchbase . (2019). *OpenBCI, Inc* Retrieved from https://www.crunchbase.com/organization/openbci-section-overview

[hbe2179-bib-0022] CyborgArts . (2019). *Cyborg Arts* Retrieved from https://www.cyborgarts.com/

[hbe2179-bib-0023] CyborgFoundation . (2019). *Cyborg Foundation* Retrieved from https://www.cyborgfoundation.com/

[hbe2179-bib-0024] CyborgFutures . (2016). *Cyborg Futures* Retrieved from http://www.cyborgfutures.com/

[hbe2179-bib-0025] CyborgNest . (2018). *A mindware company* Retrieved from https://www.cyborgnest.net/

[hbe2179-bib-0026] Davidson, R. J. , Kabat‐Zinn, J. , Schumacher, J. , Rosenkranz, M. , Muller, D. , Santorelli, S. F. , … Sheridan, J. F. (2003). Alterations in brain and immune function produced by mindfulness meditation. Psychosomatic Medicine, 65(4), 564–570. 10.1097/01.psy.0000077505.67574.e3 12883106

[hbe2179-bib-0027] Deuschl, G. , Schade‐Brittinger, C. , Krack, P. , Volkmann, J. , Schäfer, H. , Bötzel, K. , … Voges, J. (2006). A randomized trial of deep‐brain stimulation for Parkinson's disease. New England Journal of Medicine, 355(9), 896–908. 10.1056/NEJMoa060281 16943402

[hbe2179-bib-0028] Dikker, S. , & Oostrik, M. (2019). *The mutual wave projects* Retrieved from http://www.suzannedikker.net/art-science-education

[hbe2179-bib-0029] Dockery, C. A. , Hueckel‐Weng, R. , Birbaumer, N. , & Plewnia, C. (2009). Enhancement of planning ability by transcranial direct current stimulation. Journal of Neuroscience, 29(22), 7271–7277. 10.1523/jneurosci.0065-09.2009 19494149 PMC6666475

[hbe2179-bib-0030] DreamLab . (2018). *Engineering dreams* Retrieved from https://engineeringdreams.net/

[hbe2179-bib-0031] Dubljević, V. , Saigle, V. , & Racine, E. (2014). The rising tide of tDCS in the media and academic literature. Neuron, 82(4), 731–736. 10.1016/j.neuron.2014.05.003 24853934

[hbe2179-bib-0032] Eagleman, D. (2015). *Can we create new senses for humans?* TED.

[hbe2179-bib-0033] Eagleman, D. , & Novich, S. D. (2019). Methodand System for Transforming Language Inputs into Haptic Outputs, *U.S. Patent No. 20190108852* Retrieved from https://patents.justia.com/patent/20190108852

[hbe2179-bib-0034] Ebisu, A ., Hashizume, S ., Suzuki, K. , Ishii, A. , Sakashita, M. , & Ochiai, Y . (2017). *Stimulated percussions: Method to control human for learning music by using electrical muscle stimulation*. Paper presented at the Proceedings of the 8th Augmented Human International Conference New York, NY.

[hbe2179-bib-0035] Egner, T. , & Gruzelier, J. H. (2003). Ecological validity of neurofeedback: Modulation of slow wave EEG enhances musical performance. Neuroreport, 14(9), 1221–1224. 12824763 10.1097/01.wnr.0000081875.45938.d1

[hbe2179-bib-0036] Emmerson, S. (2018). In Emmerson S. (Ed.), The Routledge research companion to electronic music: Reaching out with technology. Routledge: Taylor & Francis Group.

[hbe2179-bib-0037] Emotiv . (2019). *About us* Retrieved from https://www.emotiv.com/about-emotiv/

[hbe2179-bib-0038] Ericsson . (2015). *Ten hot consumer trends for 2016* Retrieved from https://www.ericsson.com/en/trends-and-insights/consumerlab/consumer-insights/reports/10-hot-consumer-trends-for-2016

[hbe2179-bib-0039] ETH‐Zürich . (2019a). *Brain‐computer interface (BCI) race* Retrieved from https://cybathlon.ethz.ch/races-and-disciplines/bci-race.html

[hbe2179-bib-0040] ETH‐Zürich . (2019b). *Functional electrical stimulation (FES) bike race* Retrieved from https://cybathlon.ethz.ch/races-and-disciplines/fes-race.html

[hbe2179-bib-0041] Fenn, J. (2007). Understanding Gartner's hype cycles. Retrieved from https://www.gartner.com/en/documents/509085/understanding-gartner-s-hype-cycles-2007

[hbe2179-bib-0042] Fernández, T. , Herrera, W. , Harmony, T. , Díaz‐Comas, L. , Santiago, E. , Sánchez, L. , … Valdés, P. (2003). EEG and behavioral changes following neurofeedback treatment in learning disabled children. Clinical Electroencephalography, 34(3), 145–152. 10.1177/155005940303400308 14521276

[hbe2179-bib-0043] Fregni, F. , Boggio, P. S. , Nitsche, M. , Bermpohl, F. , Antal, A. , Feredoes, E. , … Pascual‐Leone, A. (2005). Anodal transcranial direct current stimulation of prefrontal cortex enhances working memory. Experimental Brain Research, 166(1), 23–30. 10.1007/s00221-005-2334-6 15999258

[hbe2179-bib-0044] Gage, G. (2015). *How to control someone else's arm with your brain* Retrieved from https://www.youtube.com/watch?time_continue=312&v=rSQNi5sAwuc

[hbe2179-bib-0045] Garcia, G . (2015). *The woman who can feel every earthquake in the world* Retrieved from http://www.hopesandfears.com/hopes/future/technology/216729-the-woman-who-can-feel-every-earthquake-in-the-world.html

[hbe2179-bib-0046] Gartry, L . (2015). *‘Cyborg activist’ Neil Harbisson, with antenna in skull, opens up on visit to Perth's Curtin University* Retrieved from https://www.abc.net.au/news/2015-08-10/cyborg-man-with-antenna-in-skull-neil-harbisson-visits-perth/6686764

[hbe2179-bib-0047] Gruzelier, J. H. (2014). EEG‐neurofeedback for optimising performance. II: Creativity, the performing arts and ecological validity. Neuroscience & Biobehavioral Reviews, 44, 142–158. 10.1016/j.neubiorev.2013.11.004 24239853

[hbe2179-bib-0048] Guger, C. , Allison, B. , Spataro, R. , Bella, V. L. , Kammerhofer, A. , Guttmann, F. , … Cho, W. (2017, October 5–8). *MindBEAGLE—A new system for the assessment and communication with patients with disorders of consciousness and complete locked‐in syndrom*. Paper presented at the 2017 IEEE International Conference on Systems, Man, and Cybernetics (SMC).

[hbe2179-bib-0049] Hampson, R. E. , Gerhardt, G. A. , Marmarelis, V. , Song, D. , Opris, I. , Santos, L. , … Deadwyler, S. A. (2012). Facilitation and restoration of cognitive function in primate prefrontal cortex by a neuroprosthesis that utilizes minicolumn‐specific neural firing. Journal of Neural Engineering, 9(5), 056012 10.1088/1741-2560/9/5/056012 22976769 PMC3505670

[hbe2179-bib-0050] Hechter, M. , & Opp, K.‐D. (2005). Social norms. New York: Russell Sage Foundation.

[hbe2179-bib-0051] Hertrich, S . (2019). *Prostheses for instincts* Retrieved from http://www.susannahertrich.com/work/prostheses-for-instincts/

[hbe2179-bib-0052] House, W. F. (1976). Cochlear implants. Annals of Otology, Rhinology & Laryngology, 85(3_suppl), 3–3. 10.1177/00034894760850s303 779582

[hbe2179-bib-0053] Huang, L. , Deng, Y. , Zheng, X. , & Liu, Y. (2019). Transcranial direct current stimulation with halo sport enhances repeated Sprint cycling and cognitive performance. Frontiers in Physiology, 10(118), 1–7. 10.3389/fphys.2019.00118 PMC6383107 30837893

[hbe2179-bib-0054] Jeffries, S. (2014). Neil Harbisson: The world's first cyborg artist. *The Guardian* Retrieved from https://www.theguardian.com/artanddesign/2014/may/06/neil-harbisson-worlds-first-cyborg-artist

[hbe2179-bib-0055] Jiang, Y. , Abiri, R. , & Zhao, X. (2017). Tuning up the old brain with new tricks: Attention training via Neurofeedback. Frontiers in Aging Neuroscience, 9(52), 1–9. 10.3389/fnagi.2017.00052 PMC5346575 28348527

[hbe2179-bib-0056] Kapur, A. , Kapur, S. , & Maes, P . (2018). *AlterEgo: A personalized wearable silent speech interface*. Paper presented at the 23rd International Conference on Intelligent User Interfaces, Tokyo, Japan.

[hbe2179-bib-0057] Kapural, L. , Deer, T. , Yakovlev, A. , Bensitel, T. , Hayek, S. , Pyles, S. , … Zovkic, P. (2010). Technical aspects of spinal cord stimulation for managing chronic visceral abdominal pain: The results from the National Survey. Pain Medicine, 11(5), 685–691. 10.1111/j.1526-4637.2010.00806.x 20210868

[hbe2179-bib-0058] Kober, S. E. , Schweiger, D. , Witte, M. , Reichert, J. L. , Grieshofer, P. , Neuper, C. , & Wood, G. (2015). Specific effects of EEG based neurofeedback training on memory functions in post‐stroke victims. Journal of Neuroengineering and Rehabilitation, 12(1), 107 10.1186/s12984-015-0105-6 26625906 PMC4666277

[hbe2179-bib-0059] Lancel, M. (2019). *E.E.G. KISS* Retrieved freom https://www.lancelmaat.nl/work/e.e.g-kiss/

[hbe2179-bib-0060] Laubacher, M. , Aksoz, E. A. , Bersch, I. , & Hunt, K. J. (2017). The road to Cybathlon 2016—Functional electrical stimulation cycling Team IRPT/SPZ. European Journal of Translational Myology, 27(4), 7086 10.4081/ejtm.2017.7086 29299220 PMC5745389

[hbe2179-bib-0061] Levy, T. J. , & Beaty, J. D. (2011). Revolutionizing prosthetics: Neuroscience framework. Johns Hopkins APL Technical Digest, 30, 223–229.

[hbe2179-bib-0062] Lomas, T. , Ivtzan, I. , & Fu, C. H. (2015). A systematic review of the neurophysiology of mindfulness on EEG oscillations. Neuroscience and Biobehavioral Reviews, 57, 401–410. 10.1016/j.neubiorev.2015.09.018 26441373

[hbe2179-bib-0063] Loo, C. K. , Alonzo, A. , Martin, D. , Mitchell, P. B. , Galvez, V. , & Sachdev, P. (2012). Transcranial direct current stimulation for depression: 3‐Week, randomised, sham‐controlled trial. British Journal of Psychiatry, 200(1), 52–59. 10.1192/bjp.bp.111.097634 22215866

[hbe2179-bib-0064] Luo, Y. H.‐L. , & da Cruz, L. (2016). The Argus® II retinal prosthesis system. Progress in Retinal and Eye Research, 50, 89–107. 10.1016/j.preteyeres.2015.09.003 26404104

[hbe2179-bib-0065] May, C. , Rapley, T. , Mair, F. S. , Treweek, S. , Murray, E. , Ballini, L. , … Finch, T. L. (2015). Normalization process theory on‐line users' manual. *Toolkit and NoMAD Instrument* Retrieved from http://www.normalizationprocess.org

[hbe2179-bib-0066] McLeod, S. (2018). *Abnormal psychology* Retrieved from https://www.simplypsychology.org/abnormal-psychology.html

[hbe2179-bib-0067] Meinzer, M. , Jähnigen, S. , Copland, D. A. , Darkow, R. , Grittner, U. , Avirame, K. , … Flöel, A. (2014). Transcranial direct current stimulation over multiple days improves learning and maintenance of a novel vocabulary. Cortex, 50, 137–147. 10.1016/j.cortex.2013.07.013 23988131

[hbe2179-bib-0068] Metz, R . (2017). *Mind‐controlled VR game really works* Retrieved from https://www.technologyreview.com/s/608574/mind-controlled-vr-game-really-works/

[hbe2179-bib-0069] Mudry, A. , & Mills, M. (2013). The early history of the cochlear implant: A retrospective. JAMA Otolaryngology–Head & Neck Surgery, 139(5), 446–453. 10.1001/jamaoto.2013.293 23681026

[hbe2179-bib-0070] Muñoz, M. (2017). *Manel Muñoz* Retrieved from https://maneliquid.portfoliobox.net/sentidodelapressi

[hbe2179-bib-0071] Muse . (2018). *Featured research with Muse* Retrieved from https://choosemuse.com/muse-research/

[hbe2179-bib-0119] Musk, E. (2019). An integrated brain‐machine interface platform with thousands of channels. 10.1101/703801 PMC6914248 31642810

[hbe2179-bib-0072] Navarro, A. (2019). *Can brain implants treat drug addicts? Chinese doctors use eye deep brain stimulation for opioid addiction treatment* Retrieved from https://www.techtimes.com/articles/243085/20190509/can-brain-implants-treat-drug-addicts-chinese-doctors-use-eye-deep-brain-stimulation-for-opioid-addiction-treatment.htm

[hbe2179-bib-0073] Netflix . (2019). *Interactive content on Netflix* Retrieved from https://help.netflix.com/en/node/62526

[hbe2179-bib-0074] neurogalMD . (2018). *Best TDCS devices of 2018: The top five devices to consider this year* Retrieved from https://neurogal.com/neuro-blog/top-five-tdcs-devices-of-2018

[hbe2179-bib-0075] NeuroSky . (2015a). *Neurosky store* Retrieved from https://store.neurosky.com/

[hbe2179-bib-0076] NeuroSky . (2015b). *Neurosky store—The adventures of NeuroBoy* Retrieved from https://store.neurosky.com/products/the-adventures-of-neuroboy-bci-technology-demo

[hbe2179-bib-0077] NeuroSky . (2019). *NeuroSky* Retrieved from http://neurosky.com/about-neurosky/

[hbe2179-bib-0078] NeuroTechX . (2019). *NEUROTECHEDU* Retrieved from http://learn.neurotechedu.com/

[hbe2179-bib-0079] Novich, S. D. , & Eagleman, D. M. (2015). Using space and time to encode vibrotactile information: Toward an estimate of the skin's achievable throughput. Experimental Brain Research, 233(10), 2777–2788. 10.1007/s00221-015-4346-1 26080756

[hbe2179-bib-0080] OpenBCI . (2019). *OpenBCI online store* Retrieved from https://shop.openbci.com/collections/frontpage/headware

[hbe2179-bib-0081] O'Rourke, P. (2015). Can Toronto‐based InterAxon's brain‐sensing headband Muse help people relax? *Financial Post* Retrieved from https://business.financialpost.com/technology/personal-tech/toronto-based-interaxons-brain-sensing-headband-muse-aims-to-help-users-learn-to-relax

[hbe2179-bib-0082] Ortner, R. , Aloise, F. , Prückl, R. , Schettini, F. , Putz, V. , Scharinger, J. , … Guger, C. (2011). Accuracy of a P300 speller for people with motor impairments: A comparison. Clinical EEG and Neuroscience, 42(4), 214–218. 10.1177/155005941104200405 22208117

[hbe2179-bib-0083] Ortner, R. , Prueckl, R. , Putz, V. , Scharinger, J. , Bruckner, M. , Schnürer, A. , … Guger, C. (2011). Accuracy of a P300 Speller for People with Motor Impairments: A Comparison. In the official Journal of the EEG and Clinical Neuroscience Society (ECNS). 2(4), 214–218. 10.1177/155005941104200405 22208117

[hbe2179-bib-0084] Pais‐Vieira, M. , Lebedev, M. , Kunicki, C. , Wang, J. , & Nicolelis, M. A. L. (2013). A brain‐to‐brain Interface for real‐time sharing of sensorimotor information. Scientific Reports, 3, 1319 10.1038/srep01319 Retrieved from https://www.nature.com/articles/srep01319-supplementary-information 23448946 PMC3584574

[hbe2179-bib-0085] Park, S.‐B. , Sung, D. J. , Kim, B. , Kim, S. , & Han, J.‐K. (2019). Transcranial direct current stimulation of motor cortex enhances running performance. PLoS One, 14(2), e0211902 10.1371/journal.pone.0211902 30794568 PMC6386265

[hbe2179-bib-0086] Pike, M. , Ramchurn, R. , Benford, S. , & Wilson, M. L. (2016). *#Scanners: Exploring the control of adaptive films using brain‐computer interaction*. Paper presented at the proceedings of the 2016 CHI Conference on Human Factors in Computing Systems, San Jose, CA.

[hbe2179-bib-0087] Puzzlebox . (2012). *Puzzlebox glass goggles* Retrieved from https://puzzlebox.io/post/project/glass-goggles/

[hbe2179-bib-0088] Quito, A . (2016). *This woman, a self‐described cyborg, can sense every earthquake in real time* Retrieved from https://qz.com/677218/this-woman-a-self-described-cyborg-can-sense-every-earthquake-in-real-time/

[hbe2179-bib-0089] Ramakrishnan, A. , Ifft, P. J. , Pais‐Vieira, M. , Byun, Y. W. , Zhuang, K. Z. , Lebedev, M. A. , & Nicolelis, M. A. L. (2015). Computing arm movements with a monkey Brainet. Scientific Reports, 5, 10767 10.1038/srep10767, https://www.nature.com/articles/srep10767-supplementary-information 26158523 PMC4497496

[hbe2179-bib-0090] Reddit . (2019). *r/tDCS* Retrieved from https://www.reddit.com/r/tDCS/

[hbe2179-bib-0091] Ribas, M . (2015). *Searching for my sense* Retrieved from https://www.youtube.com/watch?v=qU6UPUlbmLw

[hbe2179-bib-0092] Riener, R. (2016). The Cybathlon promotes the development of assistive technology for people with physical disabilities. Journal of Neuroengineering and Rehabilitation, 13(1), 49 10.1186/s12984-016-0157-2 27246601 PMC4886429

[hbe2179-bib-0093] Robertson, A . (2017). *I hacked my body for a future that never came* Retrieved from https://www.theverge.com/2017/7/21/15999544/biohacking-finger-magnet-human-augmentation-loss

[hbe2179-bib-0094] Rogers, E. M. (2003). Diffusion of innovations (Vol. 5th ed.). London, England: Free Press.

[hbe2179-bib-0095] Sarmiento, C. , San‐Juan, D. , & Prasath, S. (2016). Brief history of transcranial direct current stimulation (tDCS): from electric fishes to microcontrollers. Psychological Medicine, 46(15), 3259–3261. 10.1017/s0033291716001926 27572999

[hbe2179-bib-0096] Schultz, P. W. , Nolan, J. M. , Cialdini, R. B. , Goldstein, N. J. , & Griskevicius, V. (2007). The constructive, destructive, and reconstructive power of social norms. Psychological Science, 18(5), 429–434. 10.1111/j.1467-9280.2007.01917.x 17576283

[hbe2179-bib-0097] SecondSight . (2013). *Second Sight medical products receives FDA approval for Argus II System* [Press release]. Retrieved from http://investors.secondsight.com/news-releases/news-release-details/second-sight-medical-products-receives-fda-approval-argus-ii

[hbe2179-bib-0098] Smigielska, M. , & Cutellic, P. (2018). *Proteus* Retrieved from http://mariasni.com/project/proteus/

[hbe2179-bib-0099] Spector, H. (2018). Legal reasons and upgrading reasons. SSRN Electronic Journal. 10.2139/ssrn.3122502

[hbe2179-bib-0100] StarWarsScience . (2015). *The Force™ Trainer Ii: Hologram experience* Retrieved from http://www.starwarsscience.com/product/the-force-trainer-ii-hologram-experience/

[hbe2179-bib-0101] Swaine‐Simon, S . (2017). *DEF CON 24 –NeuroTechX—Introduction to brain based authentication* Retrieved from https://www.youtube.com/watch?v=frAhjtnvkqs

[hbe2179-bib-0102] Swan, M. (2013). The quantified self: Fundamental disruption in big data science and biological discovery. Big Data, 1(2), 85–99. 10.1089/big.2012.0002 27442063

[hbe2179-bib-0103] Thaddeus‐Johns, J. (2017). Meet the first humans to sense where north is. *The Guardian* Retrieved from https://www.theguardian.com/technology/2017/jan/06/first-humans-sense-where-north-is-cyborg-gadget

[hbe2179-bib-0104] TheThoughtEmporium . (2017). *The complete guide to magnet implants* Retrieved from https://www.youtube.com/watch?v=3aVwvJn7vpo

[hbe2179-bib-0105] TotaltDCS . (2019). *5 Best tDCS Devices of 2019* Retrieved from https://totaltdcs.com/recommended-tdcs-devices/

[hbe2179-bib-0107] Tyler, W. J. , Boasso, A. M. , Mortimore, H. M. , Silva, R. S. , Charlesworth, J. D. , Marlin, M. A. , … Pal, S. K. (2015). Transdermal neuromodulation of noradrenergic activity suppresses psychophysiological and biochemical stress responses in humans. Scientific Reports, 5, 13865 10.1038/srep13865 Retrieved from https://www.nature.com/articles/srep13865-supplementary-information 26353920 PMC4564766

[hbe2179-bib-0108] UnicornBI . (2019a). *About us* Retrieved from https://www.unicorn-bi.com/about/

[hbe2179-bib-0109] UnicornBI . (2019b). *Agent Unicorn by Anouk Wipprecht* Retrieved from https://www.unicorn-bi.com/agent-unicorn/

[hbe2179-bib-0110] Urban, T . (2017). *Neuralink and the Brain's magical future* Retrieved from https://waitbutwhy.com/2017/04/neuralink.html

[hbe2179-bib-0111] Warwick, K. (2019a). *Project Cyborg 1.0* Retrieved from http://www.kevinwarwick.com/project-cyborg-1-0/

[hbe2179-bib-0112] Warwick, K. (2019b). *Project Cyborg 2.0* Retrieved from http://www.kevinwarwick.com/project-cyborg-2-0/

[hbe2179-bib-0113] Waters‐Metenier, S. , Husain, M. , Wiestler, T. , & Diedrichsen, J. (2014). Bihemispheric transcranial direct current stimulation enhances effector‐independent representations of motor synergy and sequence learning. The Journal of Neuroscience, 34(3), 1037–1050. 10.1523/jneurosci.2282-13.2014 24431461 PMC3891947

[hbe2179-bib-0114] Weiland, J. D. , & Humayun, M. S. (2014). Retinal prosthesis. IEEE Transactions on Biomedical Engineering, 61(5), 1412–1424. 10.1109/TBME.2014.2314733 24710817 PMC4356127

[hbe2179-bib-0115] Wexler, A. (2016). The practices of do‐it‐yourself brain stimulation: Implications for ethical considerations and regulatory proposals. Journal of Medical Ethics, 42(4), 211–215. 10.1136/medethics-2015-102704 26324456

[hbe2179-bib-0116] Wexler, A. (2017). The social context of “do‐it‐yourself” brain stimulation: Neurohackers, biohackers, and Lifehackers. Frontiers in Human Neuroscience, 11(224), 1–6. 10.3389/fnhum.2017.00224 28539877 PMC5423946

[hbe2179-bib-0117] Wickson, F. , & Carew, A. L. (2014). Quality criteria and indicators for responsible research and innovation: Learning from transdisciplinarity. Journal of Responsible Innovation, 1(3), 254–273. 10.1080/23299460.2014.963004

[hbe2179-bib-0118] Worthington, R. (1982). The social control of technology. By David Collingridge. (New York: St. Martin's Press, 1980. Pp. i + 200. $22.50.). American Political Science Review, 76(1), 134–135. 10.1017/S0003055400186265

